# Intention to whistleblow: Perception of reporting skill mediates the predicting role of class consciousness and perceived probability of revenge

**DOI:** 10.12688/f1000research.142265.1

**Published:** 2023-12-06

**Authors:** Juneman Abraham, Christian Jeremia Mangapul, Destasya Nurcahyani Amaniputri, Rudi Hartono Manurung, Wing Ispurwanto

**Affiliations:** 1 Psychology Department, Faculty of Humanities, Bina Nusantara University, Jakarta, DKI Jakarta, 11480, Indonesia; 2 Japanese Department, Faculty of Humanities, Bina Nusantara University, Jakarta, DKI Jakarta, 11480, Indonesia

**Keywords:** corruption, whistleblowing intention, reporting skill, retaliation, class consciousness

## Abstract

**Background:**

A number of corruption cases would never have been revealed without the role of the whistleblower. Whistleblowers - as people who know about corruption incidents in their environment - are social capital in preventing and eradicating corruption. For this reason, it is urgent to know the configuration of psychological predictors of a person’s intention to carry out whistleblowing.

**Methods:**

Predictive correlational design with a mediation analysis was used in this study. The participants of this study were 374 Indonesians (187 males, 187 females; *M*_age_ = 25.61 years old; *SD*_age_ = 6.78 years).

**Results:**

The results showed that perception of reporting skill can mediate the predicting relationship between class consciousness, perceived probability of revenge, and intention to blow the whistle.

**Conclusions:**

Class consciousness and perceived probability of retaliation might encourage someone to feel competent to blow the whistle - or improve their reporting skill - to carry out whistleblowing.

## Introduction

The prevalence of corruption in business is a serious issue that may harm both the company’s finances and society at large. According to Castro and Phillips (2020), corporate corruption is the abuse of official power for personal gain. They also stress that it hinders national growth and undermines effective governance. Bribery, nepotism, and misuse of insider knowledge are just a few of the various ways that corruption in businesses can manifest itself ( Argandoña, 2001).

Whistleblowing is considered a potential instrument in the fight against corruption. Salihu (2019) emphasized the benefits of Nigeria’s whistleblowing policy, which led to the identification and recovery of stolen public assets as well as the conviction of offenders. Aruzzi (2019) discussed the implementation of whistleblower systems as a means to discourage and uncover corruption within governmental organizations in Indonesia. Carr and Lewis (2010) examined the potential of employment law to fight corruption by imposing obligations on those within the workplace to report corrupt activities and protecting whistleblowers.

When disclosures are made with an accurate intention and adhere to certain communication requirements when addressing matters of public interest, whistleblowing can be justified ( Kumar and Santoro, 2017). Lewis *et al.* (2014) claimed that scholars and policymakers need to better understand the intricacies and effects of whistleblowing since it is an essential procedure for holding institutions accountable.

The goal of this study is to develop a model that, using a combination of social psychological characteristics, predicts whistleblower intention. Based on the idea of planned behavior, this study draws the assumption that whistleblowing intention is an immediate antecedent of whistleblowing activity ( Ajzen, 2020). The degree to which a person has made deliberate arrangements to engage in or refrain from engaging in a certain future activity is known as intention, or behavioral intention ( Davis and Warshaw, 1992; Warshaw and Davis, 1985).

Nevertheless, the formation of the intention is not simple. Particularly in cases where there is no legal protection, whistleblowers frequently experience personal risk and harsh reprisal. When whistleblowers feel that they have more power and support, they are more likely to be successful in exposing wrongdoing ( Miceli and Near 2002). Strong proof and successful whistleblowing go hand in hand ( Apaza and Chang, 2011). Whistleblowers must possess specialized knowledge, access, and skill, according to UNODC (n.d.), that enables them to identify corruption or other serious issues that could otherwise go unnoticed. According to MacNab and Worthley (2008), in this situation, the perception of one’s competence to perform a task was a reliable predictor of their inclination to come forward, i.e. to blow the whistle.

Fear of reprisal is negatively connected to whistleblowing intents, particularly when it comes to vengeance or retaliation ( Dhamija and Rai, 2018; Khan *et al.*, 2022). If they perceive a larger fear of reprisal, whistleblowers are more likely to choose to keep quiet. Positive impressions such as professional identity, ethical orientation, and supervisor trust were dependent on employees’ fear of reprisals in a company ( Yang and Xu, 2020).

Paradoxically, then, **a person could be motivated to become more competent or skilled at making whistleblower reports if they believe that retaliation is likely to happen**. Several study findings form the basis for this idea. One may also want to counterblow if they believe that others would retaliate unfairly as a result of their whistleblowing ( Kim and Smith, 1993). In this instance, seeking retribution may be a way for a whistleblower to attain (self-)justice in situations when institutionalized advocacies from authorities are scarce ( Jäggi and Kliewer, 2016). Whistleblowers need to have more than just bare-bones competence in order to exact “moral, sweet revenge” ( Gert, 2020). A whistleblower’s drive to succeed in a later activity that supports their competence can be increased by prior competence dissatisfaction ( Fang *et al.*, 2018). People who exaggerate their performance do so in response to a threat to their performance ( Wakeman *et al.*, 2019). According to the cognitive dissonance and self-affirmation theories, a person can resolve the discrepancy between their true and false selves by developing their capacities ( Aronson *et al.*, 1999). In conclusion, people may be motivated to become more skilled or competent whistleblowers in order to anticipate or manage reprisal if they perceive retaliation or revenge to be likely.

By affecting the trade-off between justice and loyalty, class consciousness may also be a motivator for whistleblowing ( Dungan *et al.*, 2015). According to Crossley (2013), class consciousness—the recognition of oneself (“the proletariat”) as a class and of one’s collective strength—can inspire a revolution against “the bourgeoisie” and maybe thwart corruption. The relative comparisons a person draws between their socioeconomic circumstances and those of other groups are connected to this awareness ( Gartrell, 1987; Stacks, 1984).

Anvari (2019) offers a comprehensive theory of whistleblowing that incorporates social identity theorizing. This theory explains when and how social identities and types of power influence group members to report ingroup misconduct by using whistleblowing. The whistleblowing process may be motivated by collective identities and power dynamics, and it may also play a significant role in the control of moral and legal behavior, according to the model. Marxist classics have long underlined the significance of this issue, and Feng-wei’s (2006) conclusions that a systematic cultural mechanism is required to resist corruption and promote incorruptibility support the entire logic that emphasizes the relevance of class consciousness.

Whistleblowing, according to Couto *et al.* (2020), can be justified as an ethical duty to assist the group, motivated by solidarity and responsibility for the other. In other words, class consciousness can motivate political activity ( Portes, 1971); in the case of the current study, this political action is to expose corruption. Because of this, a company employee who is aware of their class must not become mired in a false consciousness that would hinder them from comprehending their shared interest in standing up to their exploiters ( Ishfaq *et al.*, 2021).

According to the preceding explanation, perceived reporting competence (1) is predicted by class consciousness and the likelihood of retaliation, and (2) predicts the desire to report wrongdoing. Perceived reporting competency may act as a mediator between the two variables’ impacts on whistleblowing intention in an integrative theoretical framework. The aim of this study is to investigate the mediation hypothesis, which claims that perceived reporting abilities can mediate the link between class consciousness and intention to report corruption as well as between perceived likelihood of retaliation and the intention.

## Methods

### Study design

The design of this study was quantitative, predictive correlational. The research was conducted in an online setting and was a cross-sectional study, so no follow-up procedure was applied. Recruitment of participants for this research was carried out from June 2020. At that time, there was a quite prominent case in Indonesia which was the trigger for this research, namely that a whistleblower in a corruption case was made a tax suspect and blackmailed by prosecutors ( Tempo, 2020), after various similar cases since 2014 which was recorded in the book entitled *Counterattacks against Scandal Whistleblowers: Case Studies Regarding the Challenges, Practices and Effectiveness of Whistleblowers in Indonesia* (as cited in Wahidin, 2021).

However, due to the COVID-19 pandemic, data collection occurred over several periods, and ended with comprehensive data processing in June 2023. The research for the field of corruption prevention was approved by Bina Nusantara University in 2020, which was then – because it designates a more extensive pool of data to be collected – followed by a grant proposal to the Indonesian Ministry of Research and Technology/National Research and Innovation Agency. However, the Indonesian Government, through the Ministry, realized the 2020 Budget Rationalization by deferring research funding due to the COVID-19 pandemic crisis. Thus, the funding attainment for this research on whistleblowing has been delayed and eventually been resubmitted to the Indonesian Ministry of Education, Culture, Research and Technology in the first quarter of 2023.

### Participants

The participants of this study were 374 people (187 women, 187 men; M _age_=25.61 years old; SD _age_=6.78 years) who came from a non-Western country, Indonesia, and were recruited using a convenience sampling technique.

The eligibility criteria of the samples were workers who worked in an organization or company with a minimum age of 15 years in accordance with Indonesian labor law. Based on data from the Indonesian Central Bureau of Statistics ( BPS, 2023), the number of workers referred to is 50,383,238. The number of samples, i.e. 374, slightly exceeding the minimum sample limit coming from a calculation using the Sample Size Calculator ( Calculator.net, 2022), with the following parameters: Confidence level of 95%, population size of 50,383,238 and population proportion of 36.34% (i.e. 50,383,238 divided by 138,632,511), i.e. 356.

Potential participants were approached via social media, such as Twitter, Instagram, and WhatsApp. After finding out that someone meets the eligibility criteria - either by asking directly via online messenger or by observing their social media account profile – the researchers conveyed that their voluntary participation is needed to fill out a questionnaire regarding the world of work. Participants were informed that the duration of time required to fill out this research questionnaire is a maximum of 20 minutes, and that the data obtained will be anonymized, not assessed as true or false, and only be used for research purposes. In addition, participants were also informed that the researchers would randomly give prizes to 10 participants in the form of IDR 50,000 (or USD 3.22) which would be transferred to the participant’s mobile phone number, but if they wanted to take part in this “lucky draw”, participants needed to write down their mobile phone number.

Written informed consent was obtained, with participants giving a check mark in the small box contained in a Google Form stating that they agreed that the data obtained from them is used and published as long as they were anonymized.

### Data collection

To measure whistleblowing intention, the author combines two concepts, namely intention and whistleblowing. Based on the definition of intention ( Davis and Warshaw, 1992; Warshaw and Davis, 1985), the dimensions of intention are (1) Conscious formulation of plan, (2) Specific behavior performance, and (3) Future behavior performance. Meanwhile, because one aspect of intention is specified behavior, vignettes are used (e.g. Ahmad *et al.*, 2013) to illustrate specific cases of corruption contextualized in the world of work. In order to determine the strength of whistleblowing intention, this study asks the question, for example, *"If you were in this concrete situation as an employee, how willing would you be to report this action to the leadership within one week? (INTEND TO means committing, planning with full awareness, deliberate or solid intention to act)*” (20 items) with response options ranging from *Strongly Not Intend* (scored 1) to *Strongly Intend* (scored 6). This study also combines types of whistleblowing (internal, external, formal, informal, anonymous, identified) ( Park *et al.*, 2008).

To measure the perception of reporting skill or competence, 13 items were used for 13 vignettes/scenarios, “ *Currently, the reporting competencies or skills that I have regarding this reporting is …*”, with response options ranging from *Very Low* (scored 1) to Very High (scored 6). To measure class consciousness, this study uses a scale developed by Keefer *et al.* (2015), which consists of the following five dimensions: (1) awareness of social class, (2) beliefs about the permeability of class groups, (3) perceptions of class conflict, (4) personal experience of being treated as a member of one’s class, and (5) identification with a class group (32 items). Examples of items are: “ *Social class is still an important issue in today’s society*”, “ *People in my social class are often treated unfairly by others*”. The response options ranges are from *Strongly Disagree* (score 1) to *Strongly Agree* (score 6). To measure the perceived probability of revenge or retaliation, 7 items were used for 7 vignettes/scenarios, “ *In your opinion, your chance of experiencing revenge if you report this action is* …”, with response options ranging from *Very Low* (scored 1) to *Very High* (scored 6).

A copy of the questionnaire can be found under *Extended data* ( Abraham *et al.*, 2023).

#### Data analysis

All psychological scales in the questionnaire were tested for validity and reliability with the criteria of item validity (corrected item-total correlation) of at least 0.250 and internal consistency (Cronbach’s a) of at least 0.600. The reliability and validity items of the research instrument are shown in Table 1. The *JASP 0.16.4.0 for Windows* was used to analyze the research data with correlation, regression, and mediation analysis. In the mediation analysis, the predicted variable was whistleblowing intention; the predictors were class consciousness and perceived probability of revenge; and the mediator was perception of reporting skill or competence.

**Table 1.** Reliability and item validities of the research instrument (N=374).

Variable	M	SD	SE _Mean_	n of items (before; after validation)	Cronbach’s Alpha	Item-rest Correlation (Min–Max)
Whistleblowing Intention (INT)	3.874	0.845	0.044	20; 20	0.905	0.347–0.653
Perception of Reporting Skill (SK)	3.825	1.027	0.053	13; 13	0.922	0.560–0.743
Class Consciousness (CC)	3.609	0.752	0.039	32; 19	0.875	0.358–0.617
Perceived Probability of Revenge (RV)	4.124	1.173	0.061	7; 7	0.895	0.599–0.763

The underlying data, complete questionnaire, and analysis script are openly available at https://zenodo.org/record/8327360 ( Abraham *et al.*, 2023).

## Results

Demographically, some participants were residents of DKI Jakarta province (N=119) which is the capital of Indonesia. In addition, other participants were residents of the Java Island (non-DKI Jakarta; N=235); Sulawesi Island (N=10); Sumatera Island (N=6); Kalimantan Island (N=2); and Bali Island (N=2). The educational composition of the participants is: Bachelor (N=197), High School (N=133), Master (N=21), Diploma (N=20), dan Doctor (N=3). The management/leadership level composition of the participants is: Non Management (Staff, Officer) (N=216), Lower Level Management (Supervisor) (N=59), Middle Level Management (Manager) (N=58), and Top Level Management (Chief Executive Officer, General Manager, Directors) (N=41).

The psychometric properties and descriptive statistics of the variables are shown in Table 1.

**Figure 1.**
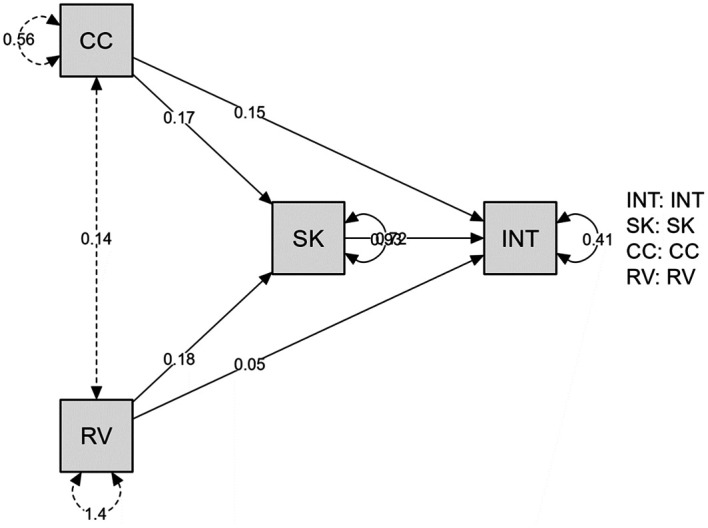
Research result. *Notes.* INT = Whistleblowing Intention; SK = Perception of Reporting Skill or Competence; CC = Class Consciousness; RV = Perceived Probability of Revenge.

The results of this study indicated that:

• All predictors, i.e. perceived reporting skills or competence ( *r*=0.755, *p*<0.001), class consciousness ( *r*=0.239, *p*<0.001), and perceived probability of revenge ( *r*=0.248, *p*<0.001), have positive correlations with whistleblowing intentions (see Table 2).
• However, regression analysis showed that while class consciousness can predict the intention ( *B*=0.151, *SE B*=0.045, *p*<0.001), perceived probability of revenge cannot directly predict it ( *B*=0.050, *SE B*=0.029, *p*>0.05 ( Table 3).
• Mediation analysis showed that class consciousness ( *B*=0.121, *SE B*=0.049, *p*<0.05) as well as perceived probability of revenge ( *B*=0.133, *SE B*=0.032, *p*<0.001) can indirectly predict the intention through perceived reporting skill or competence ( Table 4, Figure 1). Or in other words, perceived reporting skill or competence is functional as a mediator of the relationship between predictors and criterion variable.

**Table 2.** Pearson’s Correlation (N=374).

Variable		INT	SK	CC	RV	Age
1. INT	Pearson's r	—				
	p-value	—				
	Upper 95% CI	—				
	Lower 95% CI	—				
2. SK	Pearson's r	0.755 ***	—			
	p-value	<0.001	—			
	Upper 95% CI	0.796	—			
	Lower 95% CI	0.708	—			
3. CC	Pearson's r	0.239 ***	0.160 **	—		
	p-value	<0.001	0.002	—		
	Upper 95% CI	0.333	0.258	—		
	Lower 95% CI	0.141	0.060	—		
4. RV	Pearson's r	0.248 ***	0.236 ***	0.162 **	—	
	p-value	<0.001	<0.001	0.002	—	
	Upper 95% CI	0.341	0.329	0.259	—	
	Lower 95% CI	0.150	0.138	0.061	—	
5. Age	Pearson's r	0.125 *	0.016	-0.032	-0.049	—
	p-value	0.016	0.754	0.532	0.343	—
	Upper 95% CI	0.223	0.117	0.069	0.053	—
	Lower 95% CI	0.024	-0.085	-0.133	-0.150	—

*Notes.* INT = Whistleblowing Intention; SK = Perception of Reporting Skill or Competence; CC = Class Consciousness; RV = Perceived Probability of Revenge.

* *p*<0.05.

** *p*<0.01.

*** *p*<0.001.

**Table 3.** Direct predictions (N=374).

					95% Confidence Interval	
	Estimate	Std. Error	z-value	*p*	Lower	Upper
CC → INT	0.151	0.045	3.359	<0.001	0.063	0.240
RV → INT	0.050	0.029	1.704	0.088	-0.008	0.108

*Note.* Delta method standard errors, normal theory confidence intervals, ML estimator.

*Notes.* INT = Whistleblowing Intention; CC = Class Consciousness; RV = Perceived Probability of Revenge.

**Table 4.** Indirect predictions ( *N* = 374).

					95% Confidence Interval	
	Estimate	Std. Error	z-value	*p*	Lower	Upper
CC → SK → INT	0.121	0.049	2.468	0.014	0.025	0.217
RV → SK → INT	0.133	0.032	4.183	<0.001	0.071	0.195

*Note.* Delta method standard errors, normal theory confidence intervals, ML estimator.

*Notes.* INT = Whistleblowing Intention; SK = Perception of Reporting Skill or Competence; CC = Class Consciousness; RV = Perceived Probability of Revenge.

## Discussion

The current study discovered that empirical evidence supports the hypothetical model that claims perceived reporting skill or competence as a mediator that connects the predictive effects of class consciousness and perceived chance of retaliation on whistleblower intention.

Numerous studies have discovered that the perception of possible retaliation does not necessarily reduce the intention to report wrongdoing. For instance, Mesmer-Magnus and Viswesvaran (2005) claimed that the possibility of revenge (such as job loss or promotion) interacts with contextual factors (such as support from supervisors and coworkers, the size of the company, and organizational environment) to produce the choice to report wrongdoing. According to Kanojia *et al.* (2020), locus of control and perceived status/power were able to reduce the impact of retaliation on the intention of whistleblowers.

In contrast to earlier studies, the current study is looking for variables that are predicted by perceived retaliation and are also capable of increasing whistleblowing intention, such as perceived reporting competence, rather than variables that moderate the predictive effect of perceived vengeance. The indirect effect that the perception of retaliation probability has on whistleblowing intention is described below.

According to Near and Miceli (1985), a potential whistleblower faces a fundamental conundrum. If he/she reports corruption in his company and it turns out that there is a lot of it, its prevalence will be linked to poorer organizational performance. Additionally, he/she has the chance to face backlash or treachery from the business or from disgruntled employees. The company will, however, likewise drift toward anarchy and mayhem if he does not report—or forgive—so that no one takes remedial action.

Based on the research findings, perceived revenge probability does not prevent whistleblowing intents from developing; rather, it can boost efforts by enhancing reporting abilities, allowing these intentions to be carried out successfully. Borrowing insights from the world of consumer behavior, perceived betrayal is the means to understand customer retaliation ( Grégoire and Fisher, 2008). When applied in the context of a whistleblower, employees who become whistleblowers are serving the company by exposing the corruption that exists within them.

The premise of the current study is that a possible whistleblower will be willing to become a “martyr” in order to fulfill his/her noble mission to save the organization from being destroyed by perceived threats of reprisal or revenge ( Pegg, 1991; Vinten, 1997). The individual will get ready by improving his or her competence or reporting abilities so that his report does not backfire but is accepted by the organization (for follow-up) and is successful in eradicating corruption. This will ensure that this martyrdom is not in vain.

Individuals with a class consciousness, on the other hand, are aware of injustice and inequality in their surroundings and are even prepared to fight for systemic change ( Solt *et al.*, 2017). Given that critical thinking is a part of the awareness ( Kaplan, 1994), this is not shocking. Class consciousness is an effect of colonialism, according to Adenekan (2021). As a result of having been colonized by other nations (the Netherlands, England, and Japan) for several centuries, the Indonesian people are not unfamiliar with the experience of class awareness.

The awareness has an effect that significantly promotes taking corrective action to lessen inequality, particularly with those that are thought to “have a greater unfair advantage” ( Crean, 2018) — in the context of this research, those who engage in corruption — through whistleblowing. The dynamics of class consciousness, which battle against corruption by enhancing reporting abilities to safeguard working-class solidarity and sustainability, are what lead to whistleblowing intention ( Mayer, 1993).

This study’s limitation is the cross-sectional nature of the data collection, which leaves room for the possibility of common method bias to creep in. Future research should employ the longitudinal approach to lessen the inflated correlations that might result from this bias. The research’s conclusion is that additional psychological theoretical models must be developed that (1) help people feel brave or capable of overcoming the possibility of vengeance, and (2) incorporate more psycho-sociological factors like class awareness. Finding models that examine the function of factors that act as a bridge between psychological and sociological ideas is crucial since whistleblowing is neither only an individual or an organizational problem.

### Ethical considerations

This present study was approved by the Bina Nusantara University Research Committee, vide Letter Number: 149/VR.RTT/VII/2023. The ethical decree is stated in Article 1 Paragraph 2 of the Letter. Written informed consent was obtained from all participants of this study, which included consent for the research procedure to be carried out and for the publication of this article containing anonymized, analyzed, and interpreted data.

## Author roles

Juneman Abraham: Conceptualization, Data Curation, Formal Analysis, Funding Acquisition, Investigation, Methodology, Resources, Supervision, Validation, Visualization, Writing – Original Draft Preparation, Writing –Review and Editing; Christian Jeremia Mangapul: Data Curation, Formal Analysis, Investigation, Methodology, Project Administration, Resources, Software, Validation, Visualization, Writing – Original Draft Preparation; Destasya Nurcahyani Amaniputri: Data Curation, Formal Analysis, Investigation, Project Administration, Resources, Validation, Visualization, Writing – Original Draft Preparation; Rudi Hartono Manurung: Formal Analysis, Funding Acquisition, Resources, Validation, Visualization, Writing – Original Draft Preparation; Wing Ispurwanto: Conceptualization, Formal Analysis, Funding Acquisition, Project Administration, Resources, Validation, Visualization, Writing – Original Draft Preparation.

## Data Availability

Zenodo: ‘Dataset of Intention to Whistleblow: Perception of Reporting Skill Mediates the Predicting Role of Class Consciousness and Perceived Probability of Revenge’. https://zenodo.org/record/8327360 ( Abraham *et al*., 2023). This project contains the following underlying data:
- Whistleblowing Intention - Suppl Material - Data.xlsx This project contains the following extended data:
- Whistleblowing Intention - Suppl Material - Questionnaire.docx - Whistleblowing Intention - Suppl Material - Analysis Script (JASP).jasp Data are available under the terms of the Creative Commons Attribution 4.0 International license (CC-BY 4.0).
