# Supplementary material for: Intention to whistleblow: Perception of reporting skill mediates the predicting role of class consciousness and perceived probability of revenge
Source: F1000Res. 2023 Dec 6;12:1566. [Version 1] doi: 10.12688/f1000research.142265.1 (PMC10904998; doi:10.12688/f1000research.142265.1)
Supplement: Peer review history

# Reviewer report: Reviewer response for version 1

**DOI:** 10.5256/f1000research.155796.r246260

**Published:** 2024-02-29

**Authors:** Theresia Dwi Hastuti

**Affiliations:** 1 Department of Accounting, Soegijapranata Catholic University, Semarang, Indonesia

1. This article has been presented clearly and accurately. Most of the citations use recent literature, but there are several reference articles that are used from a long time ago, such as: Argandoña, 2001, Davis and Warshaw, 1992; Warshaw and Davis, 1985). Miceli and Near 2002), Kim and Smith, 1993, Aronson et al., 1999).
2. In general, the design of this research is appropriate, as well as the technical processing and analysis of research data which has been carried out well. The detailed methods and analysis provided are very likely to enable replication by other parties. However, there is statistical analysis and interpretation that needs to be added, such as the interpretation of table 1 regarding validity and reliability results. it is necessary to add the results of model testing and the ability to explain the model and its interpretation.
3.   All data sources underlying the results are available to ensure full reproducibility, but need to be updated according to local conditions. The conclusions drawn are sufficiently supported by the results obtained. This is the consistency of this research.

Is the work clearly and accurately presented and does it cite the current literature?

Yes

If applicable, is the statistical analysis and its interpretation appropriate?

Partly

Are all the source data underlying the results available to ensure full reproducibility?

Yes

Is the study design appropriate and is the work technically sound?

Yes

Are the conclusions drawn adequately supported by the results?

Yes

Are sufficient details of methods and analysis provided to allow replication by others?

Partly

Reviewer Expertise:

auditing, corporate governance, family business, SMEs, Sustainability

I confirm that I have read this submission and believe that I have an appropriate level of expertise to confirm that it is of an acceptable scientific standard.

# Reviewer report: Reviewer response for version 1

**DOI:** 10.5256/f1000research.155796.r246255

**Published:** 2024-02-21

**Authors:** Branislav Hock

**Affiliations:** 1 Centre for Cybercrime and Economic Crime, University of Portsmouth, Portsmouth, England, UK

Thank you very much for the opportunity to review this paper. The explores whistleblowing intentions as an immediate antecedent of whistleblowing activity. In doing so it attempts to develop a model, which predicts whistleblower’s intention. It is always great to see empirical work in this area.

 I am not able to comment on statistical/data part of the study.

 I suggest some improvements that will make the paper stronger.

 While the paper introduces the research problem well, it is somehow weaker in its theoretical embedding and should attempt to acknowledge the limitation of the model. Most importantly, it should further reflect more deeply on some key incentives in known whistleblowing regimes. Consider reflecting upon the following:

- transnational character of corruption, including the role of private sector firms, their compliance programmes, and “alternative” justice – [Hock B, 2022 et. al. (Ref 1)]
- Rewards – provided nationally, but also the fact that whistle-blowers are often foreign citizens being motivated by financial rewards in US and other jurisdictions. [Karpacheva E. 2024, et. al.(Ref 2)]

Consider introducing this broader set of incentives (beyond retaliation) early in the paper and revisit them later in the final discussion. Use this reflection to consider limitations of the paper and its analysis.

Is the work clearly and accurately presented and does it cite the current literature?

Partly

If applicable, is the statistical analysis and its interpretation appropriate?

I cannot comment. A qualified statistician is required.

Are all the source data underlying the results available to ensure full reproducibility?

Yes

Is the study design appropriate and is the work technically sound?

Yes

Are the conclusions drawn adequately supported by the results?

Partly

Are sufficient details of methods and analysis provided to allow replication by others?

Yes

Reviewer Expertise:

anti-corruption; economic crime

I confirm that I have read this submission and believe that I have an appropriate level of expertise to confirm that it is of an acceptable scientific standard, however I have significant reservations, as outlined above.

## References

[1] : The compliance game: Legal endogeneity in anti-bribery settlement negotiations. *International Journal of Law, Crime and Justice* .2022;71: 10.1016/j.ijlcj.2022.100560 10.1016/j.ijlcj.2022.100560

[2] : Foreign whistleblowing: the impact of US extraterritorial enforcement on anti-corruption laws in Europe. *Journal of Financial Crime* .2024;31(1) : 10.1108/JFC-11-2022-0283 1-13 10.1108/JFC-11-2022-0283

# Reviewer report: Reviewer response for version 1

**DOI:** 10.5256/f1000research.155796.r237505

**Published:** 2024-02-15

**Authors:** Karen Paul

**Affiliations:** 1 International Business, Florida International University, Miami, Florida, USA

This is an interesting article about an important topic (whistleblowing) in an understudied area of the world, so it does contribute to knowledge. The sample is not extremely rigorous but adequate. The model and discussion of meanings is adequate. I think we should have more discussion of the possible significance of Indonesian culture and behavioral norms, and I think Indonesia should be shown in the title or at least the keywords. But overall, a good job!

Is the work clearly and accurately presented and does it cite the current literature?

Yes

If applicable, is the statistical analysis and its interpretation appropriate?

Yes

Are all the source data underlying the results available to ensure full reproducibility?

Yes

Is the study design appropriate and is the work technically sound?

Yes

Are the conclusions drawn adequately supported by the results?

Yes

Are sufficient details of methods and analysis provided to allow replication by others?

Yes

Reviewer Expertise:

Business ethics, social responsibility, international business

I confirm that I have read this submission and believe that I have an appropriate level of expertise to confirm that it is of an acceptable scientific standard.
